# Validation of a Sustainable Pest Management Program to Control Coffee Berry Borer

**DOI:** 10.3390/insects17020181

**Published:** 2026-02-07

**Authors:** Pablo Benavides, Luis Eduardo Escobar, Zulma Nancy Gil, Héctor Flavio Álvarez, Hugo Mauricio Salazar, Carlos Gonzalo Mejía, Peter Follett, Hilda Diaz-Soltero

**Affiliations:** 1 National Coffee Research Center (Cenicafé), Manizales 170009, Colombia; luis.escobar@cafedecolombia.com (L.E.E.); zulma.gil@cafedecolombia.com (Z.N.G.); hector.alvarez@cafedecolombia.com (H.F.Á.); hugo.salazar@cafedecolombia.com (H.M.S.); carlosgonzalo.mejia@cafedecolombia.com (C.G.M.); 2 United States Department of Agriculture—Agricultural Research Service, Daniel K. Inouye U.S. Pacific Basin Agricultural Research Center, 64 Nowelo St., Hilo, HI 96720, USA; peter.follett@usda.gov; 3 Animal and Plant Health Inspection Service, United States Department of Agriculture, District of Columbia (USDA), Washington, DC 20250, USA

**Keywords:** biological control, coffee berry borer, cultural practices, economic analysis, *Hypothenemus hampei*, natural enemies, SPM

## Abstract

This study aimed to evaluate the effectiveness of a sustainable pest management program for controlling the coffee berry borer (CBB), *Hypothenemus hampei*, at La Catalina coffee farm (Pereira, Risaralda, Colombia) and compare it with the historical conventional control approach (2012–2022), a period during which the management of CBB was based primarily on the application of synthetic chemical insecticides. The working hypothesis was that integrating biological control agents (*Phymastichus coffea*, *Prorops nasuta*, and *Beauveria bassiana*) with cultural and monitoring practices would significantly reduce infestation levels and insecticide dependence while maintaining or improving economic profitability. From 2023 to 2024, GIS-based hotspot mapping, targeted parasitoid release, and fungal application triggered when infestation thresholds were reached were incorporated into sustainable pest management. Infestation, flight activity, and parasitism rates were monitored, and climatic variables were analyzed to determine their relationships with pest dynamics. The results showed that a sustainable pest management program reduced field infestation from a historical average of 3.3 ± 0.15% to 1.7 ± 0.2%, remaining below the 2% action threshold (F-test, *p* < 0.05). *Prorops nasuta* reduced the number of CBB life stages by 32.1%, falling from 10.9 ± 0.3 individuals per berry in non-parasitized fruits to 7.0 ± 0.7 in parasitized berries, while parasitism by *P. coffea* peaked at 70%. CBB flight activity decreased markedly compared to historical averages. The shift from a chemical approach to sustainable pest management resulted in a 26% increase in net income per hectare and a Marginal Rate of Return (MRR) of 18.06. Overall, the results confirm that a sustainable pest management program effectively suppresses CBB populations, minimizes pesticide use, and enhances the economic and environmental sustainability of coffee production systems.

## Simple Summary

The coffee berry borer (CBB) is the most destructive pest for coffee crops worldwide, reducing their yield and quality. Traditionally, some larger coffee farmers have relied on chemical insecticides to control this pest, and even under an integrated pest management (IPM) strategy, these can harm the environment and affect human health. This study tested a sustainable pest management program for CBB control at La Catalina coffee farm in Colombia, replacing chemical sprays with good agricultural practices, careful pest monitoring, and a combination of biological control agents. We used a beneficial fungus, *Beauveria bassiana*, and two types of parasitoid wasps, *Prorops nasuta* and *Phymastichus coffea*. Along with timely harvesting, we removed leftover coffee cherries from the plants and soil, and we prevented the pest from spreading during processing. These actions kept pest levels low throughout this study, even during El Niño weather conditions, which strongly favor outbreaks. This approach also increased the farmer’s income by reducing the need for expensive chemicals and additional labor, allowing for a higher coffee price due to its superior quality. This research shows that it is possible to protect coffee crops effectively without using synthetic insecticides while also protecting the environment, improving product quality, lowering production costs, and ensuring better economic returns for coffee growers.

## 1. Introduction

Agricultural producers worldwide have traditionally relied on chemical products for pest control. However, the frequency and magnitude of this practice have raised concerns about food security and ecological disruption, leading to pest outbreaks that reduce both the quantity and quality of production [1]. To address these challenges, the concept of sustainable pest management (SPM) has been introduced, promoting practices that align with natural processes, have reduced environmental impact, incorporate biological control, and enhance farmers’ livelihoods [2].

According to Savary [3], modern SPM is based on four principles: (1) biodiversity, (2) host plant resistance, (3) landscape ecology, and (4) hierarchies. These are essential for sustaining food webs and ensuring long-term crop protection as they influence plant physiology, trophic relationships, and farmer behavior. Similarly, Altieri [4] emphasized that diversified agroecosystems inherently include natural pest-control mechanisms, as their structural and management attributes directly affect herbivore dynamics.

Deguine et al. [5], in their review of SPM in cotton, highlighted that repeated use of broad-spectrum insecticides often increases pest pressure by reducing beneficial arthropod populations. In Australia, a lucerne/cotton companion planting system has been implemented to maintain the predator–pest balance and provide supplementary resources for natural enemies [5].

Molecular biotechnology has also been integrated into SPM; for example, transgenic pest-resistant varieties have been developed. Zhang et al. [6] demonstrated that cloning pest resistance genes in *Beta vulgaris* (sugar beet) increased resistance to aphids, nematodes, and root larvae, reducing production costs and providing insights into the molecular mechanisms of plant defense.

However, such approaches are difficult to apply to species like the coffee berry borer (CBB), *Hypothenemus hampei* (Ferrari) (Coleoptera: Curculionidae: Scolytinae), which lacks natural enemies outside its African center of origin [7]. According to Vega et al. [7], the CBB was first reported in Liberia in 1897 and later spread to Asia and the Americas. It is now present in nearly all coffee-producing countries, except Nepal, China, and Australia [8].

Coffee is one of the most important agricultural commodities worldwide, generating annual revenues exceeding USD 70 billion. Approximately 70% of global coffee production, estimated at over 9 million metric tons, is cultivated by smallholders on farms smaller than 5 hectares [9]. Climate change has intensified pest pressures, particularly benefiting the CBB, whose reproduction intensifies with rising temperatures [10]. Adult females infest coffee berries about 120–150 days after flowering, boring galleries into the endosperm and laying over 100 eggs. Annual economic losses due to CBBs exceed USD 500 million [7,8,11].

In Colombia, the CBB was first detected in 1988, prompting extensive research by the National Coffee Research Center (Cenicafé) on its biology, ecology, and management. This led to the implementation of integrated pest management (IPM) combining biological, cultural, and chemical strategies [12]. Bustillo [13] highlighted that promoting beneficial fauna and introducing natural enemies such as *Prorops nasuta* Waterson, 1923 (Hymenoptera: Bethylidae) and *Phymastichus coffea* LaSalle, 1990 (Hymenoptera: Eulophidae), as well as the fungus *Beauveria bassiana* (Bals.-Criv.) Vuill., could strengthen Colombia’s biological control.

Globally, initiatives such as the European Green Deal’s “Farm to Fork” strategy aim to reduce pesticide dependence by adopting sustainable pest control methods [14,15]. Consistent with these goals, Benavides et al. [16] reported up to an 81% reduction in CBB populations after releasing the parasitoid wasps *P. nasuta* and *P. coffea* on a large scale. Góngora et al. [17] emphasized the importance of integrating an ecological understanding of insect–plant interactions, climate, and habitats for more sustainable CBB management.

Other studies explored the use of predators such as *Ahasverus advena* (Walt, 1832) (Coleoptera: Cucujidae) and *Cathartus quadricollis* (Guérin-Méneville, 1844) (Coleoptera: Silvanidae), which reduced CBB populations by 63.2% and 46.2%, respectively [18]. Similarly, *Solenopsis picea* Emery, 1896, and *Crematogaster crinosa* Mayr, 1862 (Hymenoptera: Formicidae) ants exhibited predation rates of up to 78.3% and 34.3%, respectively, demonstrating strong potential as natural regulators [19]. Entomopathogenic fungi have also shown effectiveness: *B. bassiana* and *Metarhizium anisopliae* (Metschn.). Sorokīn caused 91–94% CBB mortality and reduced field infestation by 18–47% [20]. Benavides et al. [21,22] and Constantino et al. [23] demonstrated that combining timely harvesting and fruit collection with fungal application maintained infestation below 5% and increased profitability. López et al. [24] validated these findings in the department of Huila, Colombia, confirming that cultural control remains the foundation of integrated management. Economic evaluation tools, such as partial budgeting and marginal analysis, can be used to assess the profitability of these practices [25,26].

Considering the above, the objective of this study was to validate a new sustainable pest management program for controlling the coffee berry borer that combines cultural and monitoring practices, parasitoids, and fungal biopesticides and then to compare it with historical conventional control, based on the use of synthetic chemical insecticides on a large farm. We hypothesized that the SPM program to control coffee berry borer would significantly reduce infestation levels and insecticide dependence while maintaining or improving economic profitability.

## 2. Materials and Methods

### 2.1. Location and Description of the Study Area

The SPM program for controlling CBB (*Hypothenemus hampei*) was implemented at La Catalina coffee farm, located in the municipality of Pereira, Risaralda, Colombia (04°45′ N, 75°44′ W), at 1321 m above sea level. The site has an average annual temperature of 23.3 °C, a relative humidity of 79%, and annual rainfall of 2664 mm, with approximately 1665 sunshine hours/year.

The coffee farm covers 41.47 ha, of which 26.96 ha is sun-exposed coffee plantations. Of this land, 20.78 ha are used to produce certified coffee seeds. The area includes forest fragments, bamboo groves, and productive systems necessary for implementing an area-wide sustainable pest management program. The farm operates under a renewal plan under which 80% of coffee trees remain in production, and 20% are under renovation. The cultivated area includes several Arabica coffee varieties: General Castillo (38%), Cenicafé 1 (27%), Northern Castillo (11%), and smaller areas of Central Castillo, Southern Castillo, and Caturra.

### 2.2. Methodology

#### 2.2.1. Historical Conventional Control for Coffee Berry Borer at La Catalina Coffee Farm (2012–2022)

Infestation levels were evaluated after each harvest in 30 randomly selected trees using a random zig-zag sampling pattern. When infestations exceeded 2% and 50% of CBBs were penetrating the coffee berry entrance, chemical insecticides such as chlorpyrifos, fenitrothion, pirimiphos-methyl, chlorantraniliprole + thiamethoxam, or cyantraniliprole were applied.

Entomopathogenic fungi were also used, although they were often stored under inadequate conditions and without prior quality assessment. Limited operator training and poor application coverage further reduced their effectiveness.

Post-harvest control practices included closed bagging of harvested coffee and solarizing defective beans to prevent CBB dispersion. Flowering records were maintained to determine the appropriate time to spray insecticides, and trap trees were established in plots destined for renovation to reduce the dispersal of adult CBBs.

#### 2.2.2. Implementation of the Sustainable Pest Management Program (2023–2024)

##### Management of Plots by Age and Identification of Coffee Berry Borer Hotspots

A general characterization of the coffee production system at La Catalina coffee farm was conducted using Geographic Information System (GIS) tools (Software Google Earth Pro, versión 7.3). Coffee plots were classified as productive or non-productive based on their age, as recorded by the coffee farm. This information was integrated into the GIS platform to facilitate the visualization and delineation of productive areas.

The identification of CBB hotspots was conducted through an initial survey of all productive plots on the farm. Subsequently, areas with the highest pest prevalence were prioritized. These areas were characterized by a high density of branches with infested berries and infestation levels exceeding 15%. Such sites were primarily located within a border zone of five to seven rows at the plot periphery, particularly in areas adjacent to forest patches with high humidity levels and coffee weighing stations. A total of eight CBB hotspots were identified across the productive area.

In plots designated for renewal or elimination, a sanitary harvest was carried out before plant removal to reduce the risk of CBB migration to adjacent plots. Additionally, trap trees were established along plot borders to capture dispersing CBBs emerging from pruned or removed areas.

##### Evaluation of Coffee Berry Borer Infestation

Over 20 months from March 2023 to December 2024, every two weeks, 30 trees were randomly selected in the established CBB hotspots, and every month, the same number of trees were systematically chosen in the productive coffee plots, as proposed by Montoya et al. [27].

For each tree, the most productive branch in the upper third was selected, and it was visually verified that it contained more than 30 berries suitable for coffee berry borer (CBB) attack. This stage is reached approximately 120 days after flowering. On these branches, the total number of coffee berries and those attacked by CBB were counted to determine the infestation percentage. In parallel, we collected 100 infested coffee berries and recorded those with CBB adults at their entrance. The infestation levels and the position of CBB adults on coffee berries were used as decision criteria to determine the timing of *Beauveria bassiana* field applications. Applications were carried out when infestation levels exceeded 2% and more than 50% of CBB adults were in the initial berry-boring stage. In addition, the dynamics of the CBBs in the field were assessed using weighted infestation levels based on infestation percentage and coffee plot size.

To evaluate CBB infestation on the final product—the coffee parchment—weighted infestation was assessed based on the volume of harvested coffee and the calculated infestation percentage for a 200 g sample per coffee lot. This information was logged from July 2023 to November 2024, covering two main harvesting periods and one minor harvesting period.

##### Area-Wide CBB Biological Control via African Parasitoid Release

We used the same strategy described by Benavides et al. [16] to decrease the CBB dispersal rate in renovation coffee plots by releasing *Prorops nasuta*, and the colonization of young growing coffee trees by CBB was mitigated by releasing *Phymastichus coffea*. A mass release of the parasitoid *Prorops nasuta* was conducted one month before pruning to reduce the migration rate of CBB adults into neighboring plots; this species targets borers within infested coffee fruits (predatory and parasitic action). *P. nasuta* was released in fabric bags containing 500 dry parchment coffee beans parasitized by the wasps; these bags were placed along the edges and internal paths of the pruned coffee crops.

After pruning the designated plots, 1000 mature and over-ripe CBB-infested coffee berries were collected, ensuring that a range of borer stages was covered inside the fruits, allowing for evaluation of the wasp’s predatory and parasitic capacity. Those that did not contain CBBs at any stage of infestation were discarded. These berries were used to estimate the average and standard errors for the following variables within infested coffee berries: number of CBB life stages (eggs, larvae, pupae, and adults). The number of both infested and parasitized coffee berries was quantified by assessing coffee berries showing signs of predation, along with berries containing both live and dead CBBs.

Afterwards, during the critical period of coffee berry borer activity, approximately 120 days after flowering, mass release of the parasitoid wasp *Phymastichus coffea* was conducted to target CBBs in the flying adult stage on two-year-old coffee growing trees, as this species is highly effective when they begin fruit penetration. *P. coffea* was released using fabric bags containing 500 dry parchment coffee beans parasitized by the wasps. These bags were evenly distributed across each plot, ensuring complete coverage of the coffee-growing area, with priority given to zones with higher coffee berry borer activity.

To assess the impact of releasing these species in the field, monthly evaluations of borer infestation levels were conducted. For *Phymastichus coffea*, 100 CBB-infested fruits, where the adults were in entering positions, were collected biweekly to assess parasitism rates.

##### Spraying of the Entomopathogenic Fungus *Beauveria bassiana*

Optimal conditions for applying *Beauveria bassiana* were maintained through constant monitoring of infestation levels per plot and by estimating the percentage of borers at early perforation stages. At La Catalina coffee farm, this fungus was applied using commercial biopesticides after confirming that there were more than 1 × 10^9^ spores per gram, that germination occurred after 24 h (more than 90% of spores), and that purity was 95%. A concentration of at least 2 × 10^10^ spores per liter of water was sprayed during the coffee borer’s critical attack period when infestation levels exceeded 2% and when 50% of adult CBBs were entering the coffee berries. These biopesticides were applied using calibrated electric (RoyalCondor^®^-Colombia, Soacha, Colombia) and semi-stationary (Maruyama MS331 Manufactured by Maruyama Mfg. Co., Inc., Tokyo, Japan) sprayers equipped with TX4 nozzles for moderate discharge and good turbulence. Twelve days after each spraying, we evaluated the mortality caused by the fungus by dissecting 100 infested coffee berries; the value is expressed as a percentage.

##### Analysis of Historical and Current Coffee Berry Borer Flight Activity

Historical and current data on *Hypothenemus hampei* flight activity were analyzed to evaluate population dynamics over time and assess the effects of the sustainable pest management program. Historical records (2012–2022) were obtained from the La Catalina coffee farm database, based on weekly captures of adult borers in 20 alcohol-baited attractant traps distributed across the coffee plantation.

For the current period (2023–2024), the same sampling design and trap distribution were maintained to ensure comparability between datasets. The monthly and annual averages of captured borers were calculated for both the historical and current periods. These data were used to identify seasonal flight patterns, determine population peaks, and evaluate changes in pest pressure after the adoption of the SPM program.

##### Analysis of Historical and Current Climatic Variables

To determine the influence of climatic factors on CBB dynamics, historical meteorological data (2012–2024) were obtained from La Catalina coffee farm. The dataset included average temperature (°C) and monthly precipitation (mm).

Descriptive analyses were performed to examine the temporal behavior of both variables. Subsequent studies assessed potential relationships among flight activity, infestation levels, and climatic fluctuations, identifying the environmental conditions most favorable for pest development and dispersion.

##### Comparative Economic Analysis of Historical Conventional Control and Sustainable Pest Management Program for the Coffee Berry Borer

An economic analysis was performed to estimate the variable costs associated with the historical conventional control and sustainable pest management program. All values were expressed in constant U.S. dollars, deflated using the base price index (2025) published by the National Administrative Department of Statistics (DANE) as of February 2025.

The analysis focused exclusively on variable costs directly related to pest management (e.g., biological control, monitoring, and application activities), holding all other production costs ceteris paribus. Fixed fees, including taxes, administrative expenses, and financial charges, were excluded from the assessment. Both costs and revenues were standardized per hectare to facilitate comparison across management strategies and to provide a more straightforward interpretation of the economic outcomes associated with implementing the SPM program. The analysis focused on the marginal rate of return, calculated as the ratio of incremental net benefits to incremental variable costs resulting from the transition to sustainable management.

### 2.3. Statistical Analyses

Descriptive statistics were calculated for the following variables: the infestation percentage, the parasitism rate of *Phymastichus coffea*, and the historical and current flight activity of the CBB. Monthly weighted infestation data for CBBs on the La Catalina coffee farm from 2023 to 2024 under the sustainable pest management program were then compared with historical infestation records from 2012 to 2022, corresponding to the historical conventional control period when chemical insecticides were used. Mean comparisons were carried out using a contrast test (F-test) at a 5% significance level to evaluate differences between historical and sustainable CBB management strategies; similarly, to compare predation percentages of *P. nasuta*, the analyses were performed using the statistical software SAS 9.4. In addition, we conducted descriptive analyses of parasitism rates in *P. nasuta* and *P. coffea*, and monitored mortality caused by *B. bassiana* over time. Finally, we performed studies to examine potential relationships among flight activity, infestation levels, and climatic fluctuations.

## 3. Results

### 3.1. Historical Conventional Control for Coffee Berry Borer at La Catalina Coffee Farm (2012–2022)

According to historical records of annual synthetic insecticide applications for CBB management, the total insecticide use at La Catalina coffee farm was as follows: 15.9 L in 2016, 4.3 L in 2017, 2.9 L in 2018, 11.5 L in 2019, 19 L in 2020, 27.2 L in 2021, and 14.1 L in 2022. As planned, no chemical insecticides were applied during 2023 and 2024.

Following each harvest, coffee cherries were transferred into tightly sealed synthetic fiber sacks to prevent the escape of adult borers. These sacks were placed as close to the road as possible to facilitate transport to the reception hopper at midday and late afternoon. Additionally, plastic sheets coated with grease were installed over the closed reception hoppers, effectively trapping adult borers emerging from the processing coffee area. Finally, after pulping, all floating and defective beans were collected and solarized for 48 h to eliminate any remaining CBBs.

### 3.2. The Sustainable Pest Management Program (2023–2024)

#### 3.2.1. Management of Plots by Age and Identification of Coffee Berry Borer Hotspots

**Figure 1:**
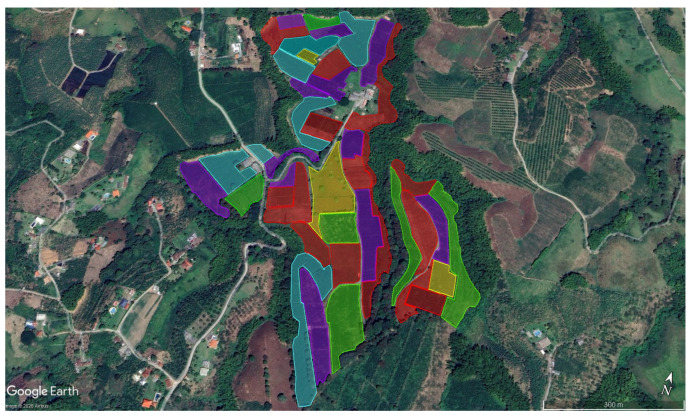
Characterization of La Catalina coffee farm. Red polygons: plots nearing elimination; purple and green polygons: plots prepared for the first and second harvests; blue and yellow polygons: remaining plots ready for the third and fourth harvests. The aerial image was obtained from Google Earth in March 2023 and January 2024.

The plots are highlighted in different colors to represent the various ages of the coffee plots on La Catalina coffee farm (Figure 1); those in red represent areas scheduled for removal or pruning.

**Figure 2:**
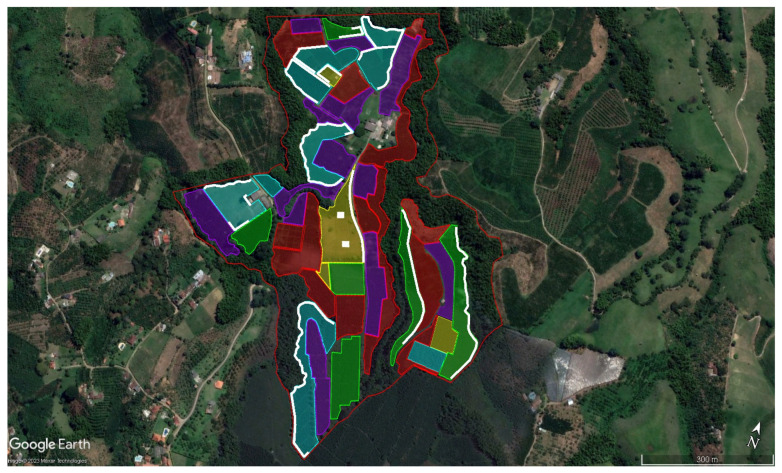
Identification and marking of hotspots (white) (areas of higher CBB concentrations) at La Catalina coffee farm. The aerial image was obtained from Google Earth in March 2023 and January 2024. Red polygons: plots nearing elimination; purple and green polygons: plots prepared for the first and second harvests; blue and yellow polygons: remaining plots ready for the third and fourth harvests.

CBB hotspots, where CBB activity was notably higher, accounted for approximately 10% of the farm’s productive coffee cultivation area and are shown as white lines (Figure 2).

#### 3.2.2. Evaluation of Coffee Berry Borer Infestation

**Figure 3:**
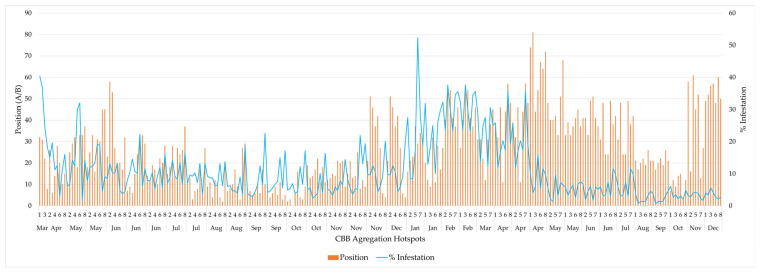
Infestation levels and CBB in entering positions on the berries in CBB aggregation hotspots.

The CBB aggregation hotspots exhibited active dynamics during the evaluation period. The highest infestation percentage of CBBs in entering positions was recorded in January 2024, with an infestation above 50%, and the highest percentage of coffee berry borers in flight was recorded in April of the same year, at slightly above 80% (Figure 3).

Between 2012 and 2022 (Group 1), the historical weighted field infestation of CBBs fluctuated between 3.4 ± 0.33% and 2.4 ± 0.12%, exceeding the action threshold of 2.0% and, in some cases, reaching the economic injury level (5.0%), as in 2016.

In contrast, during 2023 and 2024 (Group 2), following the implementation of the sustainable CBB management program, the weighted infestation remained below the 2.0% action threshold, showing statistically significant differences between the evaluated groups (Table 1 and Table 2).

**Figure 4:**
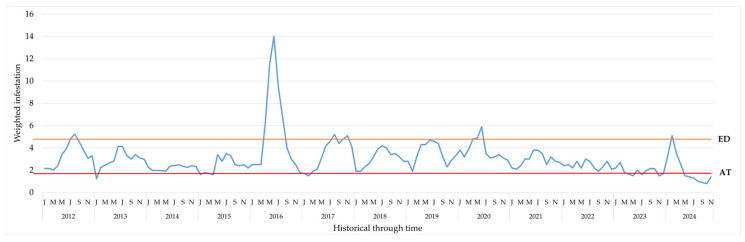
Weighted coffee berry borer infestation at La Catalina coffee farm between 2012 and 2024 (AT = action threshold 2%; EIL = economic injury level 5%).

Through monthly analysis for each year between 2012 and 2022, we observed approximately four periods in which the infestation exceeded the economic damage threshold (Figure 4). Between April 2023 and March 2024, the period considered in this research, the El Niño event occurred, yet infestation levels were significantly lower than during the last El Niño event between 2015 and 2016.

This leads us to conclude that, with the implementation of the sustainable CBB management program in 2023 and 2024, we have achieved the lowest historical recorded infestation levels at La Catalina coffee farm.

**Figure 5:**
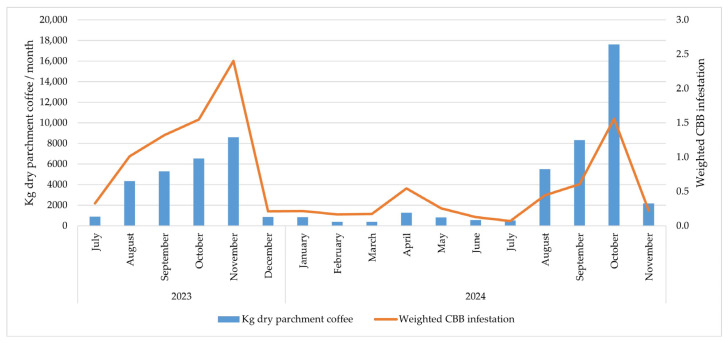
Dynamics of weighted coffee berry borer infestation in coffee parchment and monthly coffee yield (2023–2024).

In 2023, infestation levels in coffee parchment increased progressively from July (0.3%) to a peak in November (2.4%), coinciding with a rise in harvested coffee volume from 894 to 8616 kg. In September 2024, as the harvest volume increased (8323 kg), a slight surge in infestation was recorded (0.6%), reaching its highest value in October (1.6%), with 17,601 kg harvested. However, by November, infestation levels decreased again to 0.2% (Figure 5).

#### 3.2.3. Area-Wide CBB Biological Control Using African Parasitoid Release

In November 2022, *Prorops nasuta* wasps were released in the dispersal plots at La Catalina coffee farm. A total of 80,000 wet parchment coffee beans parasitized by *P. nasuta* CBBs were placed on the coffee plots, and the wasps were allowed to freely emerge and seek CBBs infesting coffee berries in the field. This material exhibited an estimated average parasitism rate of 86%, and an average of 8.7 wasps per parasitized CBB-infested bean. Thus, the number of released wasps was estimated at 598,560.

Additionally, in December 2023, another 80,000 CBB-infested beans (then parasitized) were used again in new dispersal patches. These beans had an average CBB parasitism rate of 82%, and there was an average of 6.5 wasps per bean, resulting in an estimated 426,400 wasps released.

Finally, in December 2024, 42,000 CBB-infested beans (then parasitized by *P. nasuta*) were taken to the dispersal patches. These beans had an average CBB parasitism rate of 81.2%, and there was an average of 7.8 wasp developmental stages per bean, leading to an estimated 266,011 wasps released.

After the release of *P. nasuta*, the average number of coffee berry borer stages within parasitized CBB-infested coffee berries on the coffee trap trees in dispersal plots was calculated to be 7.0 ± 0.7, compared to 10.9 ± 0.3 in non-parasitized coffee berries (Table 3); this corresponds to a final reduction of 32.1% in the coffee berry borer population in a total of 781 effective coffee berries with CBBs at different stages.

Between February and June 2023, *P. coffea* were released on 12 occasions in colonization plots at La Catalina coffee farm. During this period, a total of 112,000 humid parchment CBB-infested coffee beans, then parasitized by *P. coffea*, were placed on coffee crops. The wasps were then allowed to emerge freely and seek out flying CBB adults entering coffee berries in the field. This material had an estimated CBB parasitism rate of 64.3%, and there was an average of 4.2 coffee borers per bean. This resulted in an estimated 604,934 *P. coffea* wasps released in the field, considering that each parasitized CBB contained two wasps.

This whole procedure was repeated from January to May 2024 using a total of 118,500 humid parchment CBB-infested coffee beans parasitized by *P. coffea*. The CBB parasitism rate of these beans was 70%, with an average of 4.4 coffee borers per bean; thus, an estimated 729,960 *P. coffea* wasps were released in the field.

**Figure 6:**
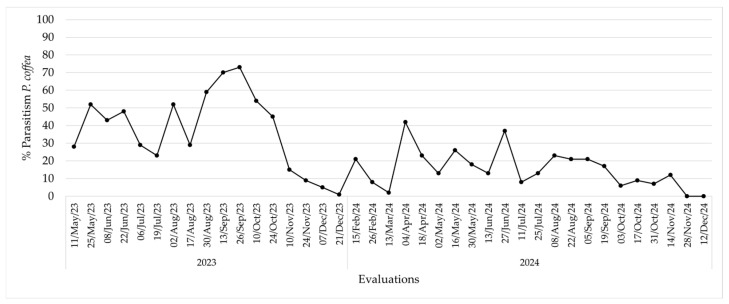
Percentage of parasitism of CBBs by *Phymastichus coffea* in colonization patches at La Catalina coffee farm in 2023 and 2024.

The percentage of parasitism of the wasp *P. coffea* in the field reached a maximum in September 2023, with values above 70%, while in 2024, the maximum value was about 40%. This percentage exhibited fluctuating dynamics and a zero parasitism level following five to six months after the final release in the field (Figure 6).

#### 3.2.4. Spraying of the Entomopathogenic Fungus *Beauveria bassiana*

La Catalina coffee farm had 23 coffee plots in production, totaling 20.88 hectares. Fungus was applied during the critical period of the CBB attack. Application was necessary when the field infestation percentage exceeded 2% and when more than 50% of the berry borers entered the coffee berries. Applications of *B. bassiana* were made in 8 of the 21 months monitored. During the remaining months, spraying was required in only one to seven coffee plots across the farm, covering no more than eight ha per application. No temporal pattern was observed that would justify calendar-based spraying. On average, both in 2023 and 2024, a single farm-wide application per year was sufficient (Table S1).

Analysis of the frequency and coverage of fungal applications in the plots at La Catalina coffee farm indicated that each plot received an average of one spraying a year. Two coffee plots required the most control with the entomopathogen—Macadamia 1 in 2023 and Costeño in 2024—both of which were sprayed up to 4 times per year during the evaluation period. Within the framework of the sustainable pest management program for *Hypothenemus hampei* at the coffee farm, each hectare of coffee crop needs to be sprayed once a year (Table 4).

#### 3.2.5. Analysis of Historical and Current Coffee Berry Borer Flight Activity

**Figure 7:**
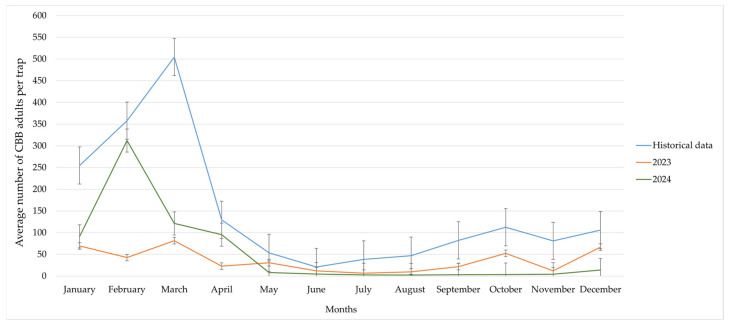
Comparison of coffee berry borer flight activity at La Catalina coffee farm in 2023–2024 with historical data (2012–2022).

Historical data (2012–2022) showed that the highest CBB adult capture rates occurred between February and March, peaking in March (504 individuals on average). The flight activity recorded in 2023 and 2024 was significantly lower across all months. In 2023, the highest flight activity occurred in March (81 individuals), but it was substantially lower than historical averages. In 2024, there was a noticeable increase in February (312), followed by consistently low values throughout the rest of the year (Figure 7).

#### 3.2.6. Analysis of Historical and Current Climatic Variables

**Figure 8:**
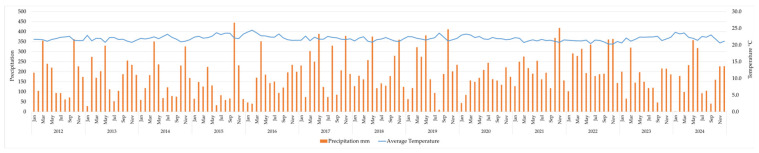
Monthly average temperature and precipitation at La Catalina coffee farm (2012–2024).

The average temperature ranged from 20.3 °C to 24.4 °C, with the highest recorded value in February 2016, coinciding with the El Niño event that year. Precipitation exhibited high variability throughout the study period, with the highest value recorded in October 2015 at close to 450 mm and the lowest in January 2024 at only 2 mm. This variability is considered a key factor in the trends and behavior of CBBs (Figure 8).

#### 3.2.7. Comparative Economic Analysis of Historical Conventional Control and Sustainable Pest Management Program for the Coffee Berry Borer

Using the partial budgeting method [25,28], the variable costs and net benefits at the end of the trial at La Catalina were calculated for both CBB management approaches. A comparison of accumulated costs under each control system showed that the sustainable pest management program incurred higher costs, mainly due to greater labor demand for activities such as harvesting, sanitization, monitoring, and trap management (Table 5).

The income generated from coffee sales under the sustainable pest management program was 26% higher than that under historical conventional control. This difference can be explained by a 2.5% increase in yield and a 20% higher selling price. Additionally, there was a 49% reduction in input costs, and no penalties were applied to the selling price, unlike in the period when chemical control was used, which showed price reductions averaging USD 82.48 per hectare.

In the marginal analysis (Table 6), considering coffee borer management strategies based on variable costs, increases in both costs and benefits (Net Income) were observed when using a sustainable pest management program rather than chemical insecticides. The sustainable pest management program yielded a marginal rate of return (MRR) of 18.06. This implies that for every USD 1.00 of incremental investment in sustainable pest management (relative to the historical conventional control), the grower not only recovers the additional cost but also obtains an extra net benefit of USD 18.06. The income generated under the sustainable pest management was 26% higher than that of the historical conventional control, driven by a 2.5% increase in productivity and a 20% higher selling price. Since agronomic management (fertilization and coffee variety) remained unchanged, these improvements are attributed to reduced berry damage and the preservation of the physical integrity of the coffee parchment.

The transition to a sustainable pest management program for CBB resulted in a 26.8% increase in net income per hectare (Table 5). Although the sustainable pest management program increased variable costs by USD 116.95/ha (primarily due to higher labor requirements for monitoring and cultural control), it generated an incremental net benefit of USD 2112.61/ha. This value yielded a Marginal Rate of Return (MRR) of 18.06 (Table 6), indicating that for every additional dollar invested in sustainable practices, the grower recovers the investment and obtains an extra net benefit of USD 18.06.

To verify the marginal analysis result, the concept of residual income was used: the difference between net benefits and the return the coffee grower would expect (residual income does not constitute profit). The highest residual income is highlighted, as determined via comparison (Table 7).

To evaluate the robustness of these findings against market fluctuations, a sensitivity analysis was performed (Table 8). The economic superiority of the sustainable pest management program proved highly resilient; the “break-even” point (where MRR = 1.0) would be reached only if labor costs increased by 422% or the coffee selling price decreased by 20.1% relative to the study period’s average. Even under a combined stress scenario (+50% labor costs and −10% prices), the MRR remained robust at 7.95, confirming the program’s financial soundness across various market conditions.

## 4. Discussion

Historical records of chemical insecticide use at La Catalina coffee farm showed an increase in use from 2019 to 2021 (up to 27.2 L), followed by a decrease in 2022 (14.1 L). CBB levels were highest during 2015–2016, when fewer chemical insecticides were used, contrasting with the significant increase in pesticide use during 2019–2021, despite climatic conditions being less favorable for borers.

These facts indicate that the appropriate timing for implementing integrated pest management (IPM) strategies was not well understood. Since 2023, the management program has been based primarily on cultural practices, the release of two African-origin parasitoids, and the spraying of the entomopathogenic fungus *B. bassiana*. This transition aligns with IPM principles and the global trend toward sustainable agricultural practices that aim to reduce dependence on chemical inputs and minimize environmental and human health risks [14].

The use of *B. bassiana* has been proven to be effective against coffee berry borers as part of the integrated management program, particularly when applied in combination with cultural measures such as harvesting fruits from the tree and the ground, reducing flying insect numbers during harvest and post-harvest by means of sealing sacks, capturing borers in a cherry collecting hopper, and managing berries and floats. Benavides and Arévalo, and Castro et al. [29,30] demonstrated that integrated management, which features timely harvesting, collection of remaining fruit, outbreak management, use of *B. bassiana*, and post-harvest practices, can reduce infestation levels and maintain a quality suitable for export, with less than 5% of coffee infested by CBBs, even under unfavorable conditions. Cultural practices are the basis of the IPM strategy.

At the La Catalina coffee farm’s processing plant, the adoption of these measures was complemented by eliminating sources of re-infestation, such as coffee pulp and floats, following the recommendations of Castro et al. [30], who demonstrated that the highest numbers of adult coffee berry borers escape from sacks opened in the field and during the drying of coffee floats. Containment measures can prevent the dispersal of up to 93% of live adults.

The use of sealed sacks and grease-impregnated covers on hoppers, as implemented at La Catalina coffee processing plant, is based on evidence that this practice significantly reduces the dispersal of berry borers from the processing area, preventing re-infestation in surrounding coffee plantations. Additionally, solarizing coffee floats eliminates insects at immature stages within coffee beans that have survived processing, a strategy validated by Castro et al. [30].

The identification of specific areas with different productive and sanitary conditions within the coffee crop demonstrated the spatial heterogeneity of CBB infestation. In this case, the plots were classified by color according to crop age and production cycle (first to fourth harvest), showing a range of productivities and management requirements. Areas marked in red represent plots undergoing plantation renewal. This differentiation is essential for managing the CBB and establishing selective management programs to optimize resources [31]. In fact, cultural practices can be readily applied to young coffee trees, and the use of sprayers is also feasible in this context. Using coffee crops older than 6 years old would decrease the efficacy of implementing any IPM measure.

During the evaluation period, coffee berry borers displayed active dynamics, with two critical peaks, one infestation in January 2024 (more than 50%) and another in April of the same year, characterized by the borers entering coffee berries (more than 80%). These peaks reflect the insect’s cyclical biology, its ability to synchronize with coffee phenological cycles, and the influence of climate on its emergence and dispersal.

These results are consistent with those reported by Benavides and Arévalo [29], who found that even under extreme conditions, high infestation hotspots can persist if strict, consistent integrated measures are not applied over time. In their study, as in this case, localized patches of high infestation were identified that required specific intervention, such as targeted harvesting, targeted application of *Beauveria bassiana*, and border demarcation with trap trees.

The trends observed in Figure 3, i.e., constant variations in the position of the borer on the fruit and the abundance of adults in flight, emphasize the importance of continuous and localized monitoring, since management strategies must be adapted not only to the infestation situation in the field but also to the specific behavior of active hotspots. As noted by Castro et al. [30], the aggregation of borers in certain locations on coffee plantations is a key characteristic that enables optimized control through targeted measures that reduce unnecessary chemical inputs.

In this study, early identification of these hotspots enabled the integration of management strategies to reduce the spread to and colonization of nearby fields. Therefore, the delimitation of these infestation hotspots and their relationship with the age and condition of the plots are key factors to consider in the integrated management of coffee berry borers, enabling timely decisions on coffee plant renewal and pruning, as well as targeted biological control. This contributes to the economic and environmental sustainability of the production system.

The significant reduction in weighted CBB infestation observed between 2023 and 2024, compared with historical records from 2012 to 2022, demonstrates the effectiveness of the SPM program in controlling coffee berry borer and highlights the overall success of sustainable pest management. In the historical period, the average infestation was 3.3%, exceeding the action threshold (2.0%) and even reaching the economic damage level in 2016 (5.4%), while in 2023 and 2024, infestation was below or equal to the action threshold, with an average of 1.7% and a statistically significant difference between groups.

The effectiveness of the sustainable management program of the coffee berry borer lies in the combination of cultural practices, the use of bio-inputs such as *Beauveria bassiana*, and ongoing monitoring of CBB populations, adjusted to the coffee’s phenological cycles and environmental conditions [17]. In this approach, the biological component does not act in isolation. However, it is enhanced by preventive and corrective measures in the field and post-harvest adopted in 2023 and 2024, such as the targeted application of *B. bassiana*, the release of the two African-origin parasitoids *P. coffea* and *P. nasuta*, and practices to prevent dispersal during post-harvest processing.

The use of *B. bassiana* in particular has been documented as an effective tool in biological control programs for CBBs, especially when applied systematically and combined with cultural practices [22]. It has been reported that applying *B. bassiana* to trees and onto the soil reduces the number of borer beetles reaching fruit by 50% and leads to a 40% mortality rate among those penetrating the fruit [22]. Furthermore, the borer beetles that survive fungal treatment lay 90% fewer eggs than in a non-sprayed control, resulting in a 55% to 75% reduction in the number of borer beetles inside the fruit [22].

Benavides et al. [22] also emphasize the importance of mitigating aggregation hotspots through targeted harvesting, trap tree management, and monitoring the insect’s position within the fruit. This is especially relevant, given that borers can emerge in mass under conditions of high humidity and stable temperatures, as occurs during periods of climate transition or phenomena such as El Niño. Through the implemented sustainable pest management program, we were able to anticipate and mitigate these phenomena, as evidenced by the absence of critical infestation levels during these two years (2023–2024).

The monthly trends in weighted CBB infestation in dry parchment coffee between July 2023 and November 2024 show a clear relationship between harvest peaks and increasing infestation levels. During the second half of 2023, a progressive increase in infestation levels was observed, peaking in November (2.4%), coinciding with the highest monthly production volume (8616 kg). A similar pattern was observed in 2024, albeit with lower infestation levels (1.6% in October) despite an even higher production level (17,601 kg), followed by a drastic reduction in November (0.2%).

This behavior is consistent with that reported by Benavides and Arévalo [29], who identified that the highest levels of infestation in dry parchment coffee tend to coincide with the main harvest peaks due to the increase in the supply of susceptible fruits and the possible emergence of remaining borers in the soil or from neighboring coffee berries in the plants. This is also supported by Constantino et al. [23], who reported intense adult CBB flight activity between collecting events during the main harvests.

However, the significant decrease observed in November 2024 could be attributed to more effective implementation of management strategies in the field during harvesting, such as the application of the fungus *B. bassiana* in complex coffee plots and hotspots following each collection of coffee berries during the main harvest of 2024. This aligns with the cultural practices applied during harvest and post-harvest, as mentioned earlier.

The successful results of the SPM program during this period can be compared with those obtained at La Finaria farm, where, after rigorous implementation of an IPM program, CBB infestation was drastically reduced in dry parchment coffee, allowing 83% of the coffee to be marketed as high-quality export coffee [29].

Furthermore, the fact that the economic injury level (5%) was not exceeded during 2024 and that the infestation remained below 2% in most months highlights the effectiveness of the sustainable approach adopted. This not only represents progress in terms of sustainability and reduction in the use of insecticides, the negative effects of which have been widely documented in the literature, but also in terms of the use of a resilient, long-term integrated management system.

Historical data (2012–2022) show that the highest CBB flight activity occurred between February and March, with a peak in the latter (504 individuals on average). This pattern is consistent with this pest’s biology, which includes emergence at adulthood, high population levels after the main harvest, and low availability of ripe fruit on trees, as well as favorable temperature and humidity conditions at the beginning of the year.

In contrast, the flight activity recorded in 2023 and 2024 was significantly lower in all months evaluated. In 2023, the highest number of captured individuals was recorded in March (81), but this was lower than the historical average. In 2024, a specific increase was observed in February (312 individuals), followed by consistently low values throughout the rest of the year. These differences suggest the positive effect of implementing this sustainable pest management program.

Decreasing flight activity reduces the dispersal of the CBB population, since adult females are responsible for colonizing new fruits [32]. It also limits re-infestation and reduces the need for control interventions, increasing the sustainability of the production system.

The climatic variability observed during the study period, with average temperatures ranging from 20.3 °C to 24.4 °C and extreme rainfall variation from 450 mm (October 2015) to just 2 mm (January 2024), is a determining factor in coffee berry borer population dynamics. In particular, the extreme temperatures and precipitation recorded during events such as El Niño (for example, in February 2016) have been identified as conditions that can accelerate the insect’s lifecycle, increasing its reproductive rate and the pressure of infestation on crops.

Coffee berry borers exhibit high ecological plasticity, with the ability to produce more generations per year in hot, dry climates, as observed during El Niño-induced drought [17]. This trend is consistent with the result documented in February 2016, when the highest temperature of the period was recorded (24.4 °C), coinciding with a significant increase in infestation recorded that same year (5.4%). Furthermore, the low precipitation recorded in January 2024 (2 mm) suggests water-stress conditions that may favor the emergence of coffee berry borer adults in search of available fruit, especially when combined with late harvests or fruit remaining permanently in the field. However, in contrast to 2016, infestation levels in 2024 remained below the action threshold (2.0%), indicating that the sustainable program implemented over the last two years has mitigated the adverse effects of climate on pest dynamics.

Under alternative climatic scenarios, such as La Niña or neutral conditions, characterized by lower temperatures and increased rainfall and generally associated with slower pest population growth, the effectiveness of the proposed sustainable pest management program is expected to be maintained or potentially enhanced.

Results from successive releases of *Prorops nasuta* in dispersal plots in 2022, 2023, and 2024 demonstrate a consistent reduction in the population density of *Hypothenemus hampei*, supporting its role as an effective component of a sustainable pest management program. The effectiveness of *P. nasuta* lies in its dual behavior as a parasitoid and predator; this bethylid attacks CBBs at all stages within the fruit, feeding on eggs and first-stage larvae and parasitizing second-stage larvae, pre-pupae, and pupae [16,33].

Accordingly, the estimated number of wasps released annually (598,560 in 2022; 426,400 in 2023; and 266,011 in 2024) was proportional to the parasitoid content per grain and the parasitism rate (>80%). Overall, these results indicate that the release densities applied were sufficient to produce a measurable suppressive effect on CBB populations under field conditions.

The 32.1% decrease in the average number of borer stages per parasitized fruit (7.0 ± 0.7) compared to un-parasitized fruit (10.9 ± 0.3) supports the effectiveness of *P. nasuta* as a predator. This finding is consistent with that reported by Benavides et al. [16], who documented reductions of up to 81% in coffee berry borer density in fallen fruit following *P. nasuta* release in dispersal plots within the context of Area-Wide biological control strategy.

Furthermore, the decrease in the number of instars per parasitized fruit suggests a reduction not only in host viability but also in host reproductive capacity. This pattern is consistent with the mode of action of *P. nasuta*, whose average lifespan of 28 days allows for sustained action in the field [16]. Unlike other introduced parasitoids, *P. nasuta* has demonstrated the ability to establish itself permanently in the Colombian coffee ecosystem, with its presence recorded on 65% of the farms evaluated and with natural parasitism levels as high as 50% in certain regions [34].

Unlike the relatively stable response observed for *P. nasuta,* the release magnitude and field persistence of *Phymastichus coffea* varied between 2023 and 2024 in the colonization plots at the La Catalina coffee farm. In 2023, with an average parasitism of 64.3% and 4.2 individual borers on average per parasitized grain, an estimated 604,934 wasps were released, while in 2024, 729,960 wasps were released due to a greater number of released grains, a higher parasitism rate (70%), and a slightly higher average number of borers per grain.

Despite the greater number of parasitoids released in 2024, inverse patterns in the field were observed in the persistence and magnitude of parasitism. In 2023, the parasitism rate exceeded 70% in September and remained relatively high for several months before declining to zero, whereas in 2024, the maximum observed rate was close to 40%, with greater fluctuations in the dynamics and an earlier disappearance of the species. This decrease in parasitism in 2024 coincides with an increase in temperature due to the El Niño event, and it appears that maximum temperatures exceeding 30 °C are not beneficial to *P. coffea.* In addition, as documented by Benavides et al. [16], *P. coffea*’s inability to establish permanently in the field is confirmed, with parasitism dropping to zero five months after the last release.

The discrepancy between the number of *P. coffea* released and their persistence in the field could be caused by abiotic and biotic factors. Benavides et al. [16] point out that climatic events such as La Niña, featuring high rainfall, can negatively affect the availability and condition of infested fruits, reducing the window for borer colonization and, therefore, the oviposition opportunities for *P. coffea*. Furthermore, the variability in the initial density of *Hypothenemus hampei* in the colonization plots and the synchrony between the release of parasitoids and the availability of CBB females entering coffee berries are decisive factors in maximizing the impact of this measure, since this species exclusively parasitizes adults during the fruit-penetration phase.

The strategy of applying entomopathogenic fungus at La Catalina coffee farm was framed within a sustainable pest management program for *Hypothenemus hampei*, with applications focused on the critical attack period, defined by infestations greater than 2% and by more than 50% of individuals positioned at coffee berry entrances. This approach aligns with Cenicafé’s recommendations to maximize efficacy and reduce unnecessary chemical inputs.

During the evaluation period (2023–2024), applications were sporadic, with a general tendency toward 1 intervention per 1 ha plot per year; the highest number was recorded in August 2023 (7 plots, 7.54 ha treated). This pattern reflects the population dynamics of the borer and their association with climatic and phenological factors, previously documented by Bustillo et al. [35], who highlighted that high humidity and rainfall stimulate mass emergence from fallen fruits.

Jaramillo et al. [20] validated the virulence effect of a mixture of different strains of *B. bassiana* and *Metarhizium anisopliae* (Metschn.). Sorokīn on the insect’s oviposition in the laboratory and on infestation and population levels in field plots. In the laboratory, the mixed strain resulted in mortality rates of 91–94%, reducing oviposition capacity by up to 87%. In the field, infestation was reduced by 18–47%. Meanwhile, Benavides et al. [21] evaluated the effects of cultural, chemical, and biological control using *B. bassiana* on coffee berry borers under field conditions, finding that cultural control was the most crucial component of IPM, leading to higher coffee production, income, and economic contribution margins.

Field efficacy is determined by the quality of the inoculum and the formulation used. Bustillo and Posada [36] established minimum standards for concentration (≈2 × 10^10^ spores L^−1^), viability (>90%), and purity (>95%) to maintain yields, and also emphasized the importance of activating the fungus on the coffee berry borer before use to preserve pathogenicity. Comparing these parameters with those used at La Catalina coffee farm would allow for identifying potential areas for improvement.

Finally, the low application frequency used in this study suggests that the monitoring and threshold system employed is effective in containing the pest with minimal intervention, reducing costs and preserving the activity of natural enemies. However, the evidence supports exploring complementary strategies, such as strain mixtures and formulations with greater persistence, to improve control during population peaks and during adverse weather conditions.

Economic analysis using the partial budget method showed that implementing the sustainable CBB management program generated a net income per hectare of USD 9981, 26% higher than under the chemical control scheme (USD 7868). This difference is mainly explained by a 2.5% increase in farm productivity and a 20% higher coffee sale price, both of which were driven by lower berry borer infestation in the coffee parchment. In contrast, in the conventional chemical system, the average penalty was USD 82.48/ha for higher coffee parchment infestation levels.

Although labor costs under the sustainable pest management program were 49% higher than under chemical management, due to greater demand for activities such as trimming, monitoring, and trap management, these costs were offset by a 49% reduction in input costs and a higher net income. Previous studies have indicated that the transition to sustainable pest management with less reliance on chemical insecticides may initially entail an increase in labor demand, but in the medium term, they generate sustainable economic and environmental benefits.

In this study, the marginal analysis yielded a rate of return of USD 18.06, meaning that for every additional USD 1 invested in switching from chemical control to sustainable pest management, the investment is recovered, and an additional benefit of USD 18.06 is obtained. This result demonstrates that investment in sustainable practices is economically viable and highly profitable, consistent with studies that have documented improvements in competitiveness and access to differentiated markets when sustainable agricultural practices are implemented free of chemical residues.

This result is significantly higher than the typical minimum acceptable marginal rate of return for agricultural technology adoption, which typically ranges from 1.5 to 2.0. Furthermore, the robustness of this profitability is confirmed by the threshold sensitivity analysis. The economic advantage of the sustainable pest management program is not a fragile outcome dependent on specific market peaks; instead, it remains the superior financial choice even under extreme stress scenarios. Specifically, sustainable pest management would remain more profitable than historical conventional control unless labor costs increased by more than 422% or the coffee selling price decreased by more than 20.1%. These safety margins suggest that the shift toward sustainable practices serves as a structural de-risking strategy for coffee production units, enhancing competitiveness by preserving grain quality rather than relying on fluctuating external premiums.

Taken together, these results support the view that using a sustainable pest management program for controlling CBB is not only environmentally friendly but also enhances profitability. However, additional challenges include resistance to pruning among farmers, limited availability of biofactories for mass rearing African parasitoids, ensuring the quality of biocontrol agents, and increased labor costs associated with these practices. To address these limitations, the use of *P. nasuta* as a specific predator to reduce CBB dispersal could be replaced by native predators such as *C. quadricollis* and *A. advena*, applied in larger numbers as suggested by Constantino et al. [37]. Moreover, *P. coffea* releases could be substituted with preventive applications of *B. bassiana* to reduce colonization in newly established coffee crops. Ensuring bioinput quality will require simple, field-friendly tests to verify the germination of *B. bassiana conidia*. Labor demands for bioinput spraying could be reduced through technologies such as uncrewed aerial vehicles (UAVs). Finally, using native predators may also help lower CBB populations on the ground, thereby decreasing labor intensity during cultural control practices.

The sustainable pest management program for coffee berry borer evaluated in this study was implemented on a coffee farm covering 41.47 ha, which demonstrates its feasibility for application at larger spatial scales under an Area-Wide management approach, as previously reported by Benavides et al. [16]. In this context, coordinated actions across contiguous coffee areas facilitate the optimization of monitoring efforts, labor organization, and biological control implementation. For smaller farms, the SPM components evaluated in this study are also adaptable. Cultural control practices and biological control can be readily implemented at the smallholder level, and in situations where access to mass-reared parasitoids is limited, native natural enemies, particularly predators, may serve as viable alternatives, as documented [18,37].

## 5. Conclusions

The results of this study support the proposed hypothesis, demonstrating that the sustainable pest management program implemented for *Hypothenemus hampei*, based on monitoring and cultural practices, the release of African-origin parasitoids *Phymastichus coffea* and *Prorops nasuta*, and spraying *B. bassiana* when needed, kept CBB infestation levels below the action threshold and below recorded historical levels, while reducing reliance on chemical insecticides. Furthermore, the program was more cost-effective than historical conventional control, showed high resilience, and reached the break-even point (MRR = 1.0), indicating its financial viability across a wide range of market conditions.

The shift from a chemical approach to a sustainable program increased net income per hectare by 26%. It yielded a marginal rate of return of 18.06, demonstrating that this sustainable pest management program is not only environmentally safer but also economically competitive, though it requires optimizing operational efficiency to offset higher labor costs.

The proposed sustainable pest management program is consistent with modern agricultural requirements, as it helps reduce insecticide residues and hazardous chemicals, supports environmental protection, and aligns with current environmental and agricultural policies. We also emphasize the need for future research to evaluate its long-term performance and broader adoption under different production systems.

## Figures and Tables

**Table 1:** Means and standard errors of the historical and current weighted field infestation.

Group	Observation	Year	Weighted Infestation %	S.E
1	1	2012	3.4	0.33
	2	2013	2.9	0.22
	3	2014	2.1	0.1
	4	2015	2.7	0.14
	5	2016	5.4	1.23
	6	2017	3.6	0.37
	7	2018	3.2	0.19
	8	2019	3.7	0.21
	9	2020	3.5	0.34
	10	2021	3.0	0.15
	11	2022	2.4	0.12
2	12	2023	1.5	0.09
	13	2024	2.0	0.38

**Table 2:** Mean and standard error for each of the evaluated groups: Group 1—historical infestation; Group 2—infestation under the sustainable pest management program to control CBB.

Group	Weighted Infestation %	S.E	Statistical Difference
1	3.3	0.15	A
2	1.7	0.2	B

Different letters indicate statistical differences at the 5% level according to the contrast test (F-test).

**Table 3:** Average number of CBB life stages (eggs, larvae, pupae, and adults) within parasitized and non-parasitized coffee berries collected from coffee trap trees in the dispersal plots.

Number (Mean ± S.E.) of CBB Life Stages Per Coffee Berry		
Coffee Berries	Mean ± (SE)	Number of Coffee Berries
Non-parasitized	10.9 ± 0.3 A	668
Parasitized	7.0 ± 0.7 B	113

Different letters indicate statistical differences at the 5% level according to the contrast test (F-test).

**Table 4:** Frequency and coverage of *Beauveria bassiana* fungus applications for coffee berry borer management at La Catalina coffee farm during the evaluation period.

Plot	ha/Plot	2023		2024	
		*B. bassiana* Applications/Year	Sprayed Hectares *	*B. bassiana* Applications/Year	Sprayed Hectares *
Costeño	1.27	3	3.81	4	5.08
Estrella 1	0.22	0	0	2	0.44
Estrella	0.58	0	0	0	0
Macadamia 1	0.97	4	3.88	1	0.97
Macadamia 3	0.85	1	0.85	2	1.70
La María	0.98	2	1.96	1	0.98
Guayacanes	1.73	0	0	0	0
Tapias	0.97	1	0.97	2	1.94
Separado	0.51	0	0	0	0
Calvario	1.8	2	3.60	1	1.80
Nogales	2.08	0	0	0	0
Tangelos	1.05	3	3.15	0	0
Alvarado	0.44	2	0.88	1	0.44
Fuete	0.37	1	0.37	0	0
Eco	1.33	0	0	2	2.66
Tanque	0.57	0	0	1	0.57
Caseta	0.83	1	0.83	1	0.83
Lote 91	0.53	0	0	1	0.53
Pulmón	0.98	1	0.98	1	0.98
Doble	1.24	1	1.24	1	1.24
Cancha	0.61	0	0	1	0.61
Aguacate	0.41	0	0	1	0.41
Cedros	0.56	0	0	0	0
Total		22	22.52	23	21.18
Average		0.95	0.98	1	0.92

* Indicates the hectares of each plot (ha/plot) multiplied by the number of fungal applications per year in that plot.

**Table 5:** Partial budget and variable costs under different coffee berry borer control systems.

	Management System	
Component	Sustainable Pest Management	Historical Conventional Control
Yield (kg DPC */ha)	2875.77	2806.11
Gross Income (USD/ha)	10,502.64	8273.08
Input Cost (USD/ha)	21.37	41.82
Labor Cost (USD/ha)	500.60	280.72
Discount due to CBB (USD/ha)	0.00	82.48
Total Variable Costs (USD/ha)	521.97	405.02
Net Income (USD/ha)	9980.67	7868.06
Gross Margin (USD/kg DPC)	3.47	2.80

* Dry Parchment Coffee.

**Table 6:** Marginal analysis of variable costs between coffee berry borer control systems.

Management Scheme	Variable Cost (USD/ha)	Net Income (USD/ha)	Marginal Cost (USD/ha)	Marginal Net Benefit (USD/ha)	Marginal Rate of Return (MRR)
Historical Conventional control	405.02	7868.06	-	-	-
Sustainable Pest Management	521.97	9980.67	116.95	2112.61	18.06

**Table 7:** Residual analysis of coffee berry borer management using a sustainable approach.

Coffee Borer Management Scheme	Costs (USD/ha)	Net Income (USD/ha)	Required Return	Residual Income
Historical Conventional control	USD 405.02	USD 7868.06	USD 60.75	USD 7807.31
Sustainable Pest Management	USD 521.97	USD 9980.67	USD 78.30	USD 9902.37

**Table 8:** Sensitivity analyses of costs.

Scenario	Variable Change	Adjusted MRR	Economic Interpretation
Baseline	Current study data	18.06	Highly Profitable
Labor Increase	+100% in labor costs	13.78	Robustly Superior
Labor Increase	+422% in labor costs	1.00	Break-even point
Price Drop	−10% in selling price	9.07	Robustly Superior
Price Drop	−20.1% in selling price	1.00	Break-even point
Combined Stress	+50% Labor & −10% Price	7.95	Superior

## Data Availability

The original contributions presented in this study are included in the article/Supplementary Materials. Further inquiries can be directed to the corresponding author.

## Supplementary Materials

The following supporting information can be downloaded at https://www.mdpi.com/article/10.3390/insects17020181/s1. Table S1: Coffee plots and hectares treated with *Beauveria bassiana* fungus for coffee berry borer control, 2023–2024.

## References

[1] Lewis W.J., Van Lenteren J.C., Phatak S.C., Tumlinson J.H. (1997). A Total System Approach to Sustainable Pest Management. Proc. Natl. Acad. Sci. USA.

[2] Velten S., Leventon J., Jager N., Newig J. (2015). What Is Sustainable Agriculture? A Systematic Review. Sustainability.

[3] Savary S., Horgan F., Willocquet L., Heong K.L. (2012). A Review of Principles for Sustainable Pest Management in Rice. Crop Prot..

[4] Altieri M.A. (1993). Ethnoscience and Biodiversity: Key Elements in the Design of Sustainable Pest Management Systems for Small Farmers in Developing Countries. Agric. Ecosyst. Environ..

[5] Deguine J.P., Ferron P., Russell D., Lichtfouse E., Navarrete M., Debaeke P., Véronique S., Alberola C. (2009). Sustainable Pest Management for Cotton Production: A Review. Sustainable Agriculture.

[6] Zhang C.L., Xu D.C., Jiang X.C., Zhou Y., Cui J., Zhang C.-X., Chen D.-F., Fowler M.R., Elliott M.C., Scott N.W. (2008). Genetic Approaches to Sustainable Pest Management in Sugar Beet (*Beta vulgaris*). Ann. Appl. Biol..

[7] Vega F.E., Infante F., Johnson A.J., Vega F.E., Hofstetter R.W. (2015). The Genus *Hypothenemus*, with Emphasis on *H. hampei*, the Coffee Berry Borer. Bark Beetles.

[8] Johnson M.A., Ruiz-Diaz C.P., Manoukis N.C., Verle Rodrigues J.C. (2020). Coffee Berry Borer (*Hypothenemus hampei*), a Global Pest of Coffee: Perspectives from Historical and Recent Invasions, and Future Priorities. Insects.

[9] Abewoy D. (2022). Impact of Coffee Berry Borer on Global Coffee Industry: Review. Int. J. Eng. Sci..

[10] Jaramillo J., Muchugu E., Vega F.E., Davis A., Borgemeister C., Chabi-Olaye A. (2011). Some Like It Hot: The Influence and Implications of Climate Change on Coffee Berry Borer (*Hypothenemus hampei*) and Coffee Production in East Africa. PLoS ONE.

[11] Lambot C., Herrera J.C., Bertrand B., Sadeghian S., Benavides P., Gaitán A. (2017). Cultivating Coffee Quality—Terroir and Agroecosystem. The Craft and Science of Coffee.

[12] Benavides P., Bustillo A.E., Cardenas R., Montoya E.C. (2003). Análisis biológico y económico del manejo integrado de la broca del café en Colombia. Rev. Cenicafé.

[13] Bustillo A.E. (2006). Una revisión sobre la broca del café, *Hypothenemus hampei* (Coleoptera: Curculionidae: Scolytinae), en Colombia. Rev. Colomb. Entomol..

[14] Fetting C. (2020). The European Green Deal.

[15] Boix-Fayos C., De Vente J. (2023). Challenges and Potential Pathways towards Sustainable Agriculture within the European Green Deal. Agric. Syst..

[16] Benavides P., Gil Z.N., Escobar L.E., Navarro-Escalante L., Follett P., Diaz-Soltero H. (2023). Pilot Testing of an Area-Wide Biological Control Strategy against the Coffee Berry Borer in Colombia Using African Parasitoids. Insects.

[17] Góngora C.E., Gil Z.N., Constantino L.M., Benavides P. (2023). Sustainable Strategies for the Control of Pests in Coffee Crops. Agronomy.

[18] Laiton L.A., Constantino L.M., Benavides P. (2018). Capacidad Depredadora de *Cathartus quadricollis* y *Ahasverus advena* (Coleoptera: Silvanidae) Sobre *Hypothenemus hampei* (Coleoptera: Curculionidae) En Laboratorio. Rev. Colomb. Entomol..

[19] Constantino L.M., Benavides P., Escobar S., Montoya J., Armbrecht I. (2022). Capacidad Depredadora de Las Hormigas *Solenopsis picea* y *Crematogaster crinosa Sobre* La Broca Del Café *Hypothenemus hampei* En Campo Con Una Solución Atrayente. Rev. Colomb. Entomol..

[20] Jaramillo J.L., Montoya E.C., Góngora C.E. (2015). *Beauveria bassiana* y *Metarhizium anisopliae* Para El Control de Broca del Café En Frutos Del Suelo. Rev. Colomb. Entomol..

[21] Benavides P., Bustillo A.E., Montoya E.C., Cárdenas R., Mejía C.G. (2002). Participación Del Control Cultural, Químico y Biológico En El Manejo de La Broca Del Café. Rev. Colomb. Entomol..

[22] Benavides P., Góngora C.E., Bustillo A.E. (2012). IPM Program to Control Coffee Berry Borer *Hypothenemus hampei*, with Emphasis on Highly Pathogenic Mixed Strains of *Beauveria bassiana*, to Overcome Insecticide Resistance in Colombia. Insecticides-Advances in Integrated Pest Management.

[23] Constantino L.M., Benavides P., Montoya E.C., Álvarez-Agudelo H.F., Trejos Pinzón J.F., Sanz-Uribe J.R. (2024). Comportamiento Poblacional y Estrategias de Control de La Broca Durante La Retención de Pases de Cosecha. Rev. Cenicafé.

[24] López-Franco F., Laiton L.A., Benavides P. (2019). Validación Del Manejo Integrado de *Hypothenemus hampei* (Ferrari) (Coleoptera: Curculionidae) En El Huila, Colombia. Rev. Cenicafé.

[25] Ávalos-Cerdas J.M., Villalobos-Monge A. (2018). Análisis Económico: Un Estudio de Caso En *Jatropha curcas* L. Mediante La Metodología de Presupuestos Parciales. Agron. Mesoam..

[26] Berger A. (2011). Calculating Unit Costs of Production and Using the Information for Enterprise Analysis and Decision Making on the Ranch. Range Beef Cow Symposium. https://digitalcommons.unl.edu/rangebeefcowsymp/282.

[27] Montoya E.C., Orozco G.L. (2005). Evaluación de Un Método de Muestreo Para Estimar La Infestación de *Hypothenemus hampei*. Rev. Cenicafé.

[28] CIMMYT (1988). La Formulación de Recomendaciones a Partir de Datos Agronómicos: Libro de Respuestas.

[29] Benavides P., Arévalo H. (2002). Manejo integrado: Una estrategia para el control de la broca del café en Colombia. Rev. Cenicafé.

[30] Castro L., Benavides P., Bustillo A.E. (1998). Dispersión y Mortalidad de *Hypothenemus hampei*, durante la recolección y beneficio del café. Manejo Integr. De Plagas (Costa Rica).

[31] Bustillo A.E. (2007). El manejo de cafetales y su relación con el control de la broca del café en Colombia. Rev. Cenicafé.

[32] Castaño A., Benavides P., Baker P.S. (2005). Dispersión de *Hypothenemus hampei* en cafetales zoqueados. Rev. Cenicafé.

[33] Bacca T., Benavides P. (2014). Evaluación de Temperaturas y Diferentes Estados Biológicos de Broca de Café Para La Cría Masiva Del Parasitoide *Prorops nasuta* (Hymenoptera: Bethylidae). Boletín Científico Cent. Mus. Mus. Hist. Nat..

[34] Maldonado C.E., Benavides P. (2007). Evaluación del establecimiento de *Cephalonomia stephanoderis* y *Prorops nasuta*, controladores de *Hypothenemus hampei*, en Colombia. Rev. Cenicafé.

[35] Bustillo A.E., Bernal M.G., Benavides P., Chaves B. (1999). Dynamics of *Beauveria Bassiana* and *Metarhizium anisopliae* Infecting *Hypothenemus hampei* (Coleoptera: Scolytidae) Populations Emerging from Fallen Coffee Berries. Fla. Entomol..

[36] Bustillo A.E., Posada F. (1996). El uso de entomopatógenos en el control de la broca del café en Colombia. Manejo Integr. Plagas.

[37] Constantino L.M., Benavides Machado P. (2023). Efecto de los depredadores *Cathartus quadricollis* y *Ahasverus advena* (Coleoptera: Silvanidae) sobre *Hypothenemus hampei* en el campo. Rev. Cenicafé.

